# Highly active antiretroviral treatment and health related quality of life in South African adults with human immunodeficiency virus infection: A cross-sectional analytical study

**DOI:** 10.1186/1471-2458-7-244

**Published:** 2007-09-14

**Authors:** Goedele M Louwagie, Max O Bachmann, Kobus Meyer, Frikkie le R Booysen, Lara R Fairall, Christo Heunis

**Affiliations:** 1 Department of Community Health, School of Health Systems and Public Health, University of Pretoria, Pretoria, South Africa; 2 School of Medicine, Health Policy & Practice, University of East Anglia, Norwich NR4 7TJ, UK; 3 Centre for Health Systems Research & Development, University of the Free State, Bloemfontein, South Africa; 4 Department of Economics/Centre for Health Systems Research & Development, University of the Free State, Bloemfontein, South Africa; 5 Knowledge Translation Unit, University of Cape Town Lung Institute, University of Cape Town, Cape Town, South Africa; 6 Centre for Health Systems Research & Development, University of the Free State, Bloemfontein, South Africa

## Abstract

**Background:**

Health Related Quality of Life (HRQoL) is an important outcome in times of Highly Active Antiretroviral Treatment (HAART). We compared the HRQoL of HIV positive patients receiving HAART with those awaiting treatment in public sector facilities in the Free State province in South Africa.

**Methods:**

A stratified random sample of 371 patients receiving or awaiting HAART were interviewed and the EuroQol-profile, EuroQol-index and Visual Analogue Scale (VAS) were compared. Independent associations between these outcomes and HAART, socio-demographic, clinical and health service variables were estimated using linear and ordinal logistic regression, adjusted for intra-clinic clustering of outcomes.

**Results:**

Patients receiving HAART reported better HRQoL for 3 of the 5 EuroQol-dimensions, for the VAS score and for the EuroQol index in bivariable analysis. They had a higher mean EuroQol index (0.11 difference, 95% confidence interval [CI] 0.04; 0.23), and were more likely to have a higher index (odds ratio 1.9, 95% CI 1.1; 1.3), compared to those awaiting HAART, in multivariate analysis. Higher mean VAS scores were reported for patients who were receiving HAART (6.5 difference, 95% CI 1.3; 11.7), were employed (9.1, 95% CI 4.3; 13.7) or were female (4.7, 95% CI 0.79; 8.5).

**Conclusion:**

HAART was associated with improved HRQoL in patients enrolled in a public sector treatment program in South Africa. Our finding that the EuroQol instrument was sensitive to HAART supports its use in future evaluation of HIV/AIDS care in South Africa. Longitudinal studies are needed to evaluate changes in individuals' HRQoL.

## Background

Globally an estimated 40.3 million people were living with HIV/AIDS by the end of 2005, with two thirds of the world's burden in sub-Saharan Africa [1]. In South Africa alone, about 5.2 million people were HIV positive in 2005 of which 520,000 had AIDS [2]. HIV/AIDS is South Africa's leading cause of death and was responsible for 38% of premature mortality by 2000 [3]. AIDS also impairs Health Related Quality of Life (HRQoL), which is an important measurable outcome of HIV treatment in the era of Highly Active Antiretroviral Treatment (HAART), complementing more objective outcomes such as death [4-8]. HAART not only reduces opportunistic infections and AIDS related mortality [9-12] but also improves HRQoL, as demonstrated repeatedly, both in developed nations and also recently in a small South African cohort treated with HAART [13-17]. The latter study demonstrated progressive improvement in HRQoL during a 12 month follow-up period in a pilot project run in a resource poor setting in the Western Cape, one of South Africa's provinces. While these findings are promising, the effectiveness of large scale HAART roll-out in South Africa is not yet known.

HRQoL can be measured with a variety of multidimensional generic and disease specific instruments, some of which have been specifically developed for people living with AIDS [8,18-21]. One commonly used generic measure is the EuroQol questionnaire, developed by the EuroQol Group. This standardized, widely validated and tested instrument provides a simple descriptive 5-dimensional profile (EQ-5D), a utility-based index of health status, and the EQ-Visual Analogue Scale (EQ-VAS) [22-29].

The EQ-5D descriptive system defines health in terms of five domains: mobility, self care, usual activities, pain or discomfort and anxiety or depression. Each domain has three levels: no problems, some/moderate problems and extreme problems. This information can be presented as an EQ-5D profile with results reported separately for each domain. Also, the 243 possible combinations of health states can be converted into a single weighted health index, the EQ index, by applying weights derived from studies among general population samples. The most widely used weights are the York tariffs, generated from a survey of 3395 adults in the United Kingdom [23,24]. In that study respondents were asked to select a length of time with perfect health that they regarded as equivalent to 10 years with a defined state of poor health [23]. Extrapolations for the 200 other possible health states were modeled mathematically [24]. Values of the EQ index can range from – 0.594 to 1, the negative values referring to a state of health regarded as worse than death. The EQ-VAS records the respondent's self-rated health status on a standard 20 cm long graduated visual analogue interval scale (from 0, the worst imaginable state of health to 100, the best imaginable state of health). Respondents rate their health state by drawing a line from the box marked "Your health state today" to the appropriate point on the EQ-VAS. A three-digit number between 000 and 100 is read off the scale from the exact point where the line crosses the scale. The EQ-VAS can be used together with the descriptive system to gain a composite picture of the respondent's health status or to track changes over time. It reflects the respondent's own assessment of his health status, in contrast to the index, which can be regarded as a societal value of the respondent's health status [30].

A review of seven generic quality-of-life instruments found that the reliability and validity of the EuroQol compared well with other instruments although it was relatively insensitive to mildly impaired health states [31]. Although not specifically designed for HIV/AIDS, this measure has also been validated, tested for reliability and successfully applied in populations living with AIDS [6,32-35]. Delate and Coons provide evidence for the construct validity of the MOS-HIV and EQ-5D in HIV positive clients [32]. They found statistically significant correlations between the EQ-5D index and HIV -1 viral loads, between EQ-5D VAS scores and CD4 cell counts, between EQ-5D VAS scores, MOS-HIV Physical Health scores and MOS-HIV Mental Health Scores, but not between EQ-5D index and CD4 cell counts. They also found moderate to strong correlations between MOS-HIV subscales and the EQ-5D index, between MOS-HIV subscales and the VAS-score and between the MOS-HIV summary score and both the as well EQ-5D index and the EQ-VAS in another clinical trial [33]. A South African study found that people living with AIDS reported a significantly lower quality of life, as measured with the EuroQol, than people without AIDS did [6]. The instrument was demonstrated to be a brief and prognostically useful predictor of mortality, hospitalization and emergency department utilization in HIV-infected adults in United States based adult HIV clinic [35]. In a recent comparative review of HRQoL measures for use in HIV/AIDS clinical trials, EQ-5D was one of the three generic outcome measures considered worthy for use in trials alongside a disease-targeted measure. Although not ideal for relatively healthy populations because of the ceiling effects, for trials with more advanced disease, the brevity of the EQ-5D was considered an advantage [36].

During 2004 HAART was introduced in South Africa's public sector HIV prevention and care program [37]. The Free State province, one of South Africa's nine provinces with high HIV burden, adapted this program to its specific needs, initially introducing a cluster of 1 hospital-based treatment site and 3 primary care assessment clinics in 4 districts and combined sites in the fifth, sparsely populated district [38]. Antiretroviral treatment services were introduced in a stepwise fashion across health districts over the course of 8 months. Patients testing positive for HIV are referred from any primary health care facility to a nurse clinician at an assessment clinic for CD4 testing and clinical evaluation. Those with a CD4 count below 200 cells/μl or WHO stage 4 are referred to the designated treatment site at a hospital for doctor's confirmation of clinical eligibility for HAART. Treatment is deferred for 2 months in patients with active tuberculosis. Patients certified as ready for treatment by the doctor, undergo a month long treatment readiness program before drug initiation. Patients are monitored on a monthly basis at the clinics, with 3 monthly review by the hospital doctor.

This study is part of a large ongoing multidisciplinary patient survey on patients' experiences of HAART, conducted by the Centre for Health Systems Research and Development of the University of the Free State. The purpose of this sub-study was to describe and compare the HRQoL of patients already receiving HAART with those awaiting HAART, for all 5 health districts. We also aimed to investigate relationships between socio-demographic, clinical, support system and health care factors and HRQoL. The study is the first of its kind to measure HRQoL in a wide-scale public sector roll-out of antiretroviral treatment in South Africa.

## Methods

### Population and sampling

This was a cross-sectional interview survey. The study population was all 1173 patients 18 years and older enrolled in the public sector HAART program in the Free State, sampled 2 months after treatment initiation of the first patients in each district. These included both patients already receiving public sector HAART and those not yet receiving HAART, but certified by a designated doctor as qualifying for treatment on the basis of CD4 counts below 200 cells/μl and/or WHO stage 4 AIDS. During this waiting period patients were required to attend the treatment readiness program. The study population excluded patients during the first 2 months of TB treatment and patients with other severe opportunistic infections which necessitated other treatment before HAART could be initiated.

A stratified random sample of patients was selected from all five districts, and from all 16 clinics providing HAART by means of anonymous patient lists obtained from all facilities. For each district 80 patients were randomly sampled, proportional to the numbers of patients per clinic and per treatment group (on HAART, awaiting HAART). In one district with fewer than 80 patients, a census of all treatment and non-treatment cases was conducted. We aimed for a sample size of 400, which provided 86% power, at the 5% significance level, to detect a 15% difference in proportions between groups (65% vs. 80%), with double as many patients on treatment as those awaiting treatment.

### Measurements

Respondents were interviewed face-to-face by trained interviewers using 2 standard paper based questionnaires – 1 for treatment and 1 for non-treatment patients, the former with additional treatment specific questions. Questions on socio-economic and demographic characteristics were taken from the 1996 South African census.

The EQ-5D-questionnaire was used to gather information on HRQoL. Subjects were also asked about socio-demographic characteristics, whether they had previously received antiretroviral treatment outside of the public sector program, recent hospitalization, smoking, alcohol and drug use and whether they had a physical or emotional caregiver or a treatment supporter. The questionnaires were translated into Afrikaans, English, Sotho, isiXhosa and Zulu, but because of patient demographics, only the Afrikaans, English and Sotho versions were administered. For the EQ-5D, best available translations into Afrikaans, isiXhosa, Zulu and Sotho, which had previously been completed by a number of Southern African researchers according to standards set by the EuroQol Group, were used [39-41]. Where possible, interviews were held at assessment clinics with home visits being conducted in cases where this was not feasible. Treatment starting dates were obtained by interviews and from patient records.

The questionnaire was piloted by two researchers among three Department of Health staff members receiving HAART in the private sector. Data quality control took place at three levels: in-field editing, site visit editing and data editing. Test-retest reliability was not assessed because it was not feasible for patients to return for a later interview within the recommended one to two weeks, solely for this purpose [42]. It was also plausible that their health states could change rapidly.

Written consent for the interviews and for access to the clinical records was obtained from all participating patients. The study was approved by the Ethics Committee of the Faculty of Humanities of the University of the Free State and was registered with the EuroQol group. Study results were treated confidentially.

### Analysis

All data analysis was performed with the statistical software package Stata Version 8.0. The researchers calculated the EQ index values for each of the respondents, weighted by the York tariffs [23,24]. Treatment duration was defined as the time from start of treatment to date of interview. Treatment duration was categorized with cut points at 0/1, 29/30, 59/60 and 89/90 days.

Descriptive statistics were used to describe the health status and other socio-demographic and behavioral characteristics of the subjects. Simple logistic, ordinal logistic and linear regressions as well non-parametric tests were used to identify potential predictors of the 5 EQ-domains, the VAS and the EQ index score and potential confounders of belonging to the HAART group vs. the non-HAART group. Simple ordinal logistic regression was used to compare EuroQol dimensions between patients on HAART and those awaiting HAART. Regression analyses were adjusted for intra-clinic clustering of outcomes using Stata's Huber-White robust sandwich estimator. Cuzick's non-parametric test for trend was used to measure trends in the index and the VAS scores across ordered treatment duration categories [43]. Spearman's rank correlation test was used to test for correlation between non-normally distributed numerical variables. The inter-item correlation of the 5 component dimensions of the EQ-5D index was estimated using Cronbach's alpha.

EQ index scores were not normally distributed and so were analyzed as follows. First, we divided the index into three ordered index categories for analysis with multiple ordinal logistic regression. All EQ index values of 1 were included in the highest category to reduce non-differential misclassification. Second, we performed bootstrapped multiple linear regression using cluster (clinic) sampling, with 1000 replications, and obtained bias-corrected accelerated percentile confidence intervals for regression coefficients [44]. For the VAS score, regression diagnostics showed that linear regression was appropriate. The explanatory variables initially used in the models with VAS and EQ index as outcomes were total personal income, sex, employment status, previous antiretroviral treatment, recent hospitalization, having a physical or emotional caregiver or a treatment buddy, education, rooms in house, age, treatment site and treatment duration or treatment group. Explanatory variables were removed from each model if their adjusted p-value was more than 0.05 and if removing them did not change the odds ratio or beta-coefficient for treatment by more than 10%. Analyses accounted for the stratified sampling by including district as a covariate, and for intra-clinic correlation of outcomes by robust error adjustment.

## Results

### Patients' demographic, socioeconomic and health characteristics

A total of 371 patients were interviewed (268 on treatment and 103 awaiting treatment). Median treatment duration, for those on treatment with known treatment duration (220 patients), was 70 (IQR 38.5–90) days.

Interviewees were young (75% aged <43 years), with nearly twice as many women as men (65% vs. 35%), largely unemployed (85%) and dependent on social grants (49%). Three quarters of respondents (74%) lived in houses with 4 or less rooms and 67% had completed 10 or less of the 12 grades of primary and secondary school (Table 1). The HAART group differed significantly from the non-HAART group in terms of socio-economic status as indicated by income (p = 0.005), housing size (p = 0.017) and employment status (p = 0.008), but not by educational level or access to grants. No respondent reported consuming cannabis, while a similarly small percentage of both groups reported alcohol consumption or smoking. Predictably, the HAART group reported having a treatment buddy significantly more often than the non-treatment group (p = 0.007). Patients already on antiretroviral treatment when accessing the program received preferential access so as to limit treatment interruptions. This is reflected in the higher proportion of previously treated patients in the HAART group (p = 0.006).

**Table 1:** Patients' demographic, socioeconomic and health characteristics

	**All respondents (N = 371)**	**Receiving HAART (N = 268)**	**Awaiting HAART (N = 103)**	P-value*
	Median (IQR)	Median (IQR)	Median (IQR)	

Age	37 (31–43)	37 (32–44)	36 (31–41)	0.17
Personal income (ZAR per month)**	740 (0–740)	740 (0–780)	740 (0–740)	0.005

	*n (%)*	*n (%)*	*n (%)*	
Female	243 (65)	176 (66)	67 (65)	0.88
Education ≤ grade 10	249 (67)	180 (67)	69 (67)	0.98
Health insurance	11 (3)	8 (3)	3 (3)	0.98
Employed	54 (15)	45 (17)	9 (9)	0.008
Receiving welfare grant	182 (49)	132 (49)	51 (50)	0.96
≤ 4 rooms in home	273 (74)	186 (69)	87 (84)	0.017
Smoked past 30 days	41 (11)	26 (10)	15 (15)	0.29
Drank alcohol past 30 days**	18 (5)	11 (4)	7 (7)	0.33
Has physical caregiver	131 (35)	90 (34)	41 (40)	0.46
Has emotional caregiver	178 (48)	123 (46)	55 (53)	0.21
Has treatment buddy	166 (45)	136 (51)	30 (29)	0.007
Previous antiretroviral treatment	25 (7)	24 (9)	1 (1)	0.006
Hospitalized since entering HAART program	37 (10)	25 (9)	12 (12)	0.62

*Simple logistic regression adjusted for intraclinic clustering of outcomes

** 1 missing value

Socio-demographic and patient support variables associated with HRQoL are presented in Table 2. Males were more likely than females to report problems in some domains. Unemployed patients were more likely than employed patients to report problems in some domains, and had significantly worse EQ index and VAS values. Patients with a physical caregiver reported a lower quality of life for all EuroQol measures.

**Table 2:** Health-related quality of life by selected socio-demographic and patient support variables

	**Male (N = 128)**	**Female (N = 243)**	**P**	**Unemployed (N = 317)**	**Employed (N = 54)**	**P**	**No Physical caregiver (N = 239)**	**Physical caregiver (N = 131)**	**P**
	*n (%)*	*n (%)*		*n (%)*	*n (%)*		*n (%)*	*n (%)*	

Mobility									
No problems	95 (74)	196 (81)	0.031*	243 (77)	48 (89)	0.15*	194 (81)	97 (74)	0.031*
Some problems	28 (22)	45 (19)		68 (21)	5 (9)		43 (18)	29 (22)	
Severe problems	5 (4)	2 (1)		6 (2)	1 (2)		2 (1)	5 (4)	
Self care									
No problems	112 (88)	225 (93)	0.23*	286 (90)	51 (94)	0.32*	224 (94)	112 (86)	0.001*
Some problems	10 (8)	14 (6)		22 (7)	2 (4)		11 (5)	13 (10)	
Severe problems	6 (5)	4 (2)		9 (3)	1 (2)		4 (2)	6 (5)	
Usual activities**									
No problems	92 (72)	197 (81)	0.034*	242 (77)	47 (87)	0.040*	195 (82)	94 (72)	0.06*
Some problems	24 (19)	37 (15)		55 (17)	6 (11)		35 (15)	25 (19)	
Severe problems	12 (9)	8 (3)		19 (6)	1 (2)		8 (3)	12 (9)	
Pain/discomfort									
None	71 (55)	136 (56)	0.84*	167 (53)	40 (74)	0.009*	143 (60)	64 (49)	0.012*
Some	47 (37)	93 (38)		126 (40)	14 (26)		89 (37)	50 (38)	
Severe	10 (8)	14 (6)		24 (8)	0 (0)		7 (3)	17 (13)	
Depression/anxiety									
None	80 (63)	168 (69)	0.28*	210 (66)	38 (70)	0.56*	170 (71)	78 (60)	0.006*
Some	34 (27)	60 (25)		82 (26)	12 (22)		54 (23)	39 (30)	
Severe	14 (11)	15 (6)		25 (8)	4 (7)		15 (6)	14 (11)	
	Median (IQR)	Median (IQR)		Median (IQR)	Median (IQR)		Median (IQR)	Median (IQR)	
Index Score	0.85 (0.69–1)	0.85 (0.73–1)	0.27^&^	0.85 (0.69–1)	1 (0.73–1)	0.027^&^	1 (0.73–1)	0.80 (0.62–1)	0.001^&^
VAS score	60 (50–80)	70 (50–80)	0.08^&^	60 (50–80)	70 (60–83)	0.003^&^	70 (50–80)	60 (50–73)	0.004^&^

* Ordinal logistic regression adjusted for intraclinic clustering of outcomes

** Information missing for 1 respondent

^&^Wilcoxon rank sum test

Percentages for some dimensions do not add up to 100% because of rounding errors

Cronbach's alpha for the EQ index was 0.85, indicating a high degree of correlation between the five dimensions. The EQ index correlated well with VAS scores (Spearman's ρ = 0.48, Pearson's R^2 ^= 0.29).

### Influence of HAART on Health Related Quality of Life

The EQ-5D results showed that patients awaiting HAART commonly reported problems, with 57% reporting that pain or discomfort caused problems, 42% reporting that depression or anxiety caused problems, 29% reporting problems with mobility, and with an EQ index of 0.69 and a VAS score of 62. Respondents on HAART were significantly less likely than those awaiting treatment to report a higher problem score for 3 of the 5 EQ domains. Their median EQ index (0.87 vs. 0.80, p = 0.04) and VAS scores (70 vs. 60, p = 0.04) were higher than for those awaiting treatment (Table 3). Secondary analysis of the data with exclusion of respondents who, at enrollment in the public sector program, had previously received antiretroviral treatment produced similar results. There was a trend of higher index scores for consecutive treatment duration groups (p = 0.03), but this was not the case for the VAS score (p = 0.29).

**Table 3:** Health-related quality of life of patients on HAART vs. those awaiting HAART

	**All respondents (N = 371)**	**Receiving HAART (N = 268)**	**Awaiting HAART (N = 103)**	**P-value**
**EQ-5 Dimensions**	n (%)	n (%)	n (%)	
Mobility				
No problems	291 (78)	218 (81)	73 (71)	0.026*
Some problems	73 (20)	48 (18)	25 (24)	
Severe problems	7 (2)	2 (1)	5 (5)	
Self care				
No problems	337 (91)	252 (94)	85 (83)	0.001*
Some problems	24 (6)	13 (5)	11 (11)	
Severe problems	10 (3)	3 (1)	7 (7)	
Usual activities**				
No problems	289 (78)	216 (81)	73 (71)	0.07*
Some problems	61 (16)	42 (16)	19 (18)	
Severe problems	20 (5)	9 (3)	11 (11)	
Pain/discomfort				
None	207 (56)	162 (60)	45 (44)	0.022*
Some	140 (38)	92 (34)	48 (47)	
Severe	24 (6)	14 (5)	10 (10)	
Depression/anxiety				
None	248 (67)	188 (70)	60 (58)	0.15*
Some	94 (25)	61 (23)	33 (32)	
Severe	29 (8)	19 (7)	10 (10)	
**Index score**				
Median (IQR)	0.85 (0.69–1)	0.87 (0.73–1)	0.80 (0.66–1)	0.004^$^
Mean (SD)	0.77 (0.33)	0.80 (0.29)	0.69 (0.40)	
**VAS score**				
Median (IQR)	65 (50–80)	70 (50–80)	60 (50–70)	0.04^$^
Mean (SD)	65 (20)	66 (20)	62 (23)	

* Ordinal logistic regression adjusted for intraclinic clustering of outcomes

** Information missing for 1 respondent

^$ ^Wilcoxon rank sum test

Percentages for some dimensions do not add up to 100% because of rounding errors

Table 4 shows the influence of HAART on the EQ index, adjusted for employment status and district and for intra-clinic clustering of outcome. Patients on HAART were nearly twice as likely to have a higher index as those awaiting HAART. Employed respondents were more likely to have a higher HRQoL than unemployed respondents, but this did not reach statistical significance. Using linear regression with bootstrapping, patients on HAART had on average a 0.11 higher index than those not on HAART while patients with a physical caregiver had a lower index score than those who did not have a physical caregiver. Similarly, being on HAART was associated with a 6.5 higher VAS score, compared to those not on HAART, independent of the positive effects of employment and being female on self-reported HRQol (Table 5).

**Table 4:** Predictors of EQ index*

**Explanatory variable**	Odds ratio**	95% CI***	P-value***
*Ordinal logistic regression model*			
On HAART vs. awaiting HAART	1.9	1.1–3.2	0.019
Employed vs. unemployed	1.7	0.91–3.2	0.10
			
**Explanatory variable**	Coefficient****	95% CI***	
*Linear regression with bootstrapping*			
On HAART vs. awaiting HAART	0.11	0.04; 0.23	-
Physical caregiver vs. none	-0.12	-0.21; -0.04	-

* Variables initially entered in model: total personal income, sex, employment status, previous antiretroviral treatment, recent hospitalization, having a physical or emotional caregiver or a treatment buddy, education, rooms in house, age, treatment site and treatment duration or treatment group

** Odds ratio of having a higher index

*** Adjusted for district and for intra-clinic clustering of outcome

**** Difference in mean index values

**Table 5:** Predictors of Visual Analogue Scale score*

**Explanatory variable**	Coefficient*	95% CI*	P-value**
On HAART vs. awaiting HAART	6.5	1.3–11.7	0.018
Employed vs. unemployed	9.1	4.3–13.7	0.001
Female vs. male	4.7	0.79–8.5	0.021

* Multiple linear regression model with the following variables initially entered in model: total personal income, sex, employment status, previous antiretroviral treatment, recent hospitalization, having a physical or emotional caregiver or a treatment buddy, education, rooms in house, age, treatment site and treatment duration or treatment group

** Adjusted for district and for intra-clinic clustering of outcome

## Discussion

This study shows independent associations between HAART and measures of HRQoL, despite the relatively short treatment duration. This was true for both the VAS score and the EQ index. This supports a cohort study of HAART patients in Khayelitsha township in the Western Cape province [34] that found improved EQ-5D after 12 months of treatment. The effect of HAART on VAS estimated in the present study (6.5 points, Table 4) was smaller than reported in the Khayelitsha study (14.4 points increase after 12 months). These two studies results are however, not directly comparable because in our study the duration of treatment was shorter, our design was cross-sectional rather than longitudinal, and we adjusted for gender and employment status whereas that study did not [34].

These findings are also in keeping with comparable European studies. Tramarin *et al*. compared the HRQoL of a historical cohort of Italian AIDS patients not on HAART, with a matched cohort on HAART, with both groups followed up for 6 months [16]. The HAART cohort had far fewer severe and total disability days than the 1994 cohort. Furthermore, the HAART cohort had significantly higher scores at the end of the 6-month follow-up in two of the Nottingham Health Profile dimensions (energy and emotional reactions), while in the non-HAART group no statistically significant changes were observed over 6 months. Similarly, Carrieri *et al*. demonstrated that a year of HAART leads to significant improvements in most dimensions of the Medical Outcome 36 item Short Form Survey [17]. In another European cohort study, the socio-demographic, biological and clinical predictors of HRQoL of 809 HIV-infected patients were assessed with multivariate models at baseline and at 6 months follow-up, using the Medical Outcome Survey-HIV [45]. Symptoms, previous hospitalization, satisfaction with information and education were associated with physical and mental health. Not taking antiretroviral therapy at the baseline was significantly associated with poor physical health in the bivariable analysis, but not after adjustment for other variables in the model.

HRQoL results are not a function of physical health state alone. Socio-economic and demographic factors, such as income, employment status, education and gender may influence self-perceived HRQoL, independent of health status [26,28,29]. In our study unemployed people had a lower VAS and somewhat lower index score than employed people. This could be because that sicker people are more likely to leave work, rather than joblessness itself affecting well-being, so longitudinal studies are needed to distinguish the direction of this effect. Unlike in other studies, income was not an independent predictor of HRQoL [29]. This could be because the main source of income of our population was in the form of disability grants. Beneficial effects of income may have been offset by higher levels of disability among grant recipients. Women rated their health higher than men on the VAS, which contrasts with a previous finding of poorer ratings of women using the World Health Organization QoL HIV questionnaire [20]. In our study, women may have less advanced stages of disease when seeking health care. Preliminary analysis of CD4 count results of patients enrolled in the Free State HAART program suggest their pretreatment CD4 counts are higher (L. Fairall, personal communication, February 2006). But HRQoL reflects a complex interaction of many factors other than health status. In a South African community based survey, the presence of a disability, an income below US$140 per month, unemployment and age in years were independent predictors of the VAS score [29]. A large UK survey found variations in health status measured with EuroQol, across social classes, age, gender, education, housing tenure and marital status [26]. Employed people and better educated people reported significantly less problems. Years of education was found to be an independent predictor of the EQ-VAS in patients with rheumatoid arthritis in the United Kingdom and employed patients had significantly higher EQ index and EQ-VAS scores than retired and unemployed people [28].

Our study had several limitations. Most importantly, as it was not a randomized trial it could be biased by unmeasured confounders. The treatment duration, for many respondents, was too short for the HAART effects to be shown. Because of the cross-sectional nature of the study, the inferences we can draw about causal relationships are limited. We could not adjust treatment effects for important clinical predictors, such as CD4 counts, viral loads and disease stage, which are known to be associated with HRQoL [17]. Waiting for HAART could also have decreased HRQoL. However, the quality of life measures were significantly different for patients receiving vs. those awaiting HAART, for three of the five EQ-5D domains, for the EQ index and for the VAS scale, with and without adjustment for confounders, so these differences are unlikely to be due to type 1 errors.

A general problem of generic quality of life measures is that they are insensitive to illnesses that are not severe. The valuations, or weightings, for the EQ index score were derived from the UK population. It is notable that those valuations depended on the age and the sex of respondents: equivalent health states among those aged 18–59 had higher valuations then those aged over 60 and men had higher valuations then women [23]. Valuations in a South African population may differ because of different socio-demographic characteristics and different cultural attitudes towards health and disease [46]. The EQ index may be better suited for economic evaluation studies than for clinical studies [30] and the EuroQol instrument does not capture all relevant dimensions of HRQoL such as social relationships, sexuality and spirituality [20]. The EQ-5D question on anxiety and depression does not distinguish between these two different constructs, which may be problematic in this context. Nonetheless, its simplicity, previous validation and use in South African populations, including in people with HIV, and comparability with other studies, made it appropriate for this study. As part of our wider research project, we plan to assess participating patients' changes in HRQoL on a 6-monthly basis over a period of at least 3 years and to complement interview data with clinical data to get a more detailed picture of factors influencing HRQoL.

## Conclusion

This cross-sectional study suggests that HAART is effective in improving people's self-reported HRQoL. Further studies are needed to assess the influence of unmeasured confounders on the results and longer term effects. The EuroQol instrument is a useful instrument to monitor AIDS related patient outcomes, in addition to clinical endpoints such as incidence of opportunistic infections and death. It is easy to measure and takes into account patients' points of view. Our finding that the EQ-5D was highly sensitive to HAART supports its use in future evaluation of HIV/AIDS care in South Africa.

## Competing interests

The author(s) declare that they have no competing interests.

## Authors' contributions

GM conceived and designed the HRQoL study, performed analysis and interpretation of data and drafted the manuscript, MOB assisted with the design of the HRQoL study, the analysis and interpretation of data and the critical review of the manuscript, KM assisted with the conception and design of questionnaires for the broader CP6 study of which this study formed a part, supervised data collection and quality control and critically reviewed the manuscript, FLRB conceived and designed the broader CP6 study and sampling procedures and critical reviewed the manuscript, LRF assisted with data analysis, translated the EuroQol according to EuroQol standards and critical reviewed the manuscript, CH assisted with the conception and design of broader CP6 study and critical reviewed the manuscript. All authors approved and read the final manuscript.

## Pre-publication history

The pre-publication history for this paper can be accessed here:


